# Predicting Potentially Fatal COVID-19 Disease in Pregnant Patients Using the Neutrophil-to-Lymphocyte Ratio (NLR)

**DOI:** 10.3390/jcm12216896

**Published:** 2023-11-02

**Authors:** Dorina Supák, Balázs Mészáros, Balázs Turi, Zoltán Herold, Zoltán Kukor, Sándor Valent

**Affiliations:** 1 Department of Obstetrics and Gynecology, Semmelweis University, 1082 Budapest, Hungary; supak.dorina@semmelweis.hu (D.S.); meszaros.balazs@semmelweis.hu (B.M.); turi.balazs@stud.semmelweis.hu (B.T.); valent.sandor@semmelweis.hu (S.V.); 2 Division of Oncology, Department of Internal Medicine and Oncology, Semmelweis University, 1082 Budapest, Hungary; herold.zoltan@semmelweis.hu; 3 Department of Molecular Biology, Institute of Biochemistry and Molecular Biology, Semmelweis University, 1094 Budapest, Hungary

**Keywords:** COVID-19, coronavirus, pregnancy, NLR, neutrophil-to-lymphocyte ratio, mechanical ventilation, prediction

## Abstract

Objective: To evaluate the neutrophil-to-lymphocyte ratio (NLR) values’ possible predictive role in fatal and severe cases of COVID-19 disease in pregnant women. Design and data collection: A case-control study was conducted with the inclusion of 45 pregnant COVID-19 patients. All the data were obtained from the hospital information system of Semmelweis University by two of the authors. Results: Statistical analyses showed that NLR values were significantly higher in patients with fatal COVID-19 compared to those who survived the disease, with or without mechanical ventilation. The study also assessed whether NLR values measured on the first day of hospitalization or at their peak provided better markers of disease severity. While both the first-day and peak NLR values were evaluated in patients who did not survive the disease, only the peak NLR values had predictive value regarding patient death. Conclusion: Based on our results, the peak NLR values appear to be useful markers of COVID-19 severity, with a cut-off value of 18.05. However, the authors suggest and hope that larger sample size studies will be conducted to further validate the findings of their research.

## 1. Introduction

Severe acute respiratory syndrome coronavirus 2 (SARS-CoV-2 or COVID-19) is a ribonucleic acid (RNA) virus that belongs to the Betacoronavirus genus, group 2 [1,2]. Even though most patients who get infected with SARS-CoV-2 experience mild symptoms or remain symptomless (80%), 15% of the infected experience moderate symptoms, and 5% develop severe symptoms [3,4]. The main risk factors for severe outcomes of COVID-19 disease are male gender, elderly age, obesity, and comorbidities, especially hypertension, diabetes, and cardiovascular diseases [5]. Studies indicate that severe COVID-19 in pregnant patients is strongly associated with pre-eclampsia, preterm birth, gestational diabetes, intensive care unit (ICU) admission, mechanical ventilation, cesarean delivery, low birth weight, and NICU admission [6].

To predict the severity of SARS-CoV-2 virus infection, various methods have been used since the occurrence of the disease [7,8,9]. One of these methods was the closer surveillance of neutrophil-to-lymphocyte ratio (NLR) [10,11], which has already been evaluated in pregnant individuals battling with COVID-19 but also in other medical conditions (e.g., pre-eclampsia, gestational diabetes mellitus, and miscarriage) [12,13,14,15]. The neutrophil-to-lymphocyte ratio is a simple and widely used biomarker, which is calculated by dividing the absolute neutrophil count (ANC) by the absolute lymphocyte count (ALC). The NLR is typically measured from a complete blood count (CBC) or blood test [16]. The NLR was proven to be a valuable marker in assessing inflammatory conditions such as autoimmune diseases and different forms of cancer [17,18,19,20]. Given that COVID-19 is characterized by a significant inflammatory component [21], it is comprehensible why researchers examined the NLR as a potential prognostic marker, recognizing its role in predictive assessments. The advantages of the usage of the NLR as a COVID-19 severity biomarker is that it can be easily applied in low-resource settings, as per the relatively low cost of peripheral blood tests [22].

In this study, we present the cases of 10 COVID-19 patients who were critically ill during their pregnancies and lost their lives due to the infection, thus evaluating whether the NLR has a prognostic value in the screening of potentially fatal COVID-19 in pregnant patients. We compared the data of the above-mentioned 10 patients with two control groups: one, which consisted of pregnant patients who also needed invasive or non-invasive ventilation but survived the COVID-19 disease, and another control group, in which the pregnant patients also survived but had moderate COVID-19 disease. During the clinical work with COVID-19 patients, our researchers had the hypothesis that those patients who face more severe outcomes due to their infections have higher NLR values on the day of the hospital admission and on the day when the NLR is peaking. Our objective was to evaluate the predictive value of the NLR in assessing the severity of illness in pregnant COVID-19 patients.

## 2. Methods

### 2.1. Study Design and Patient Selection

In this case-control study, 45 patients who received treatment at the clinics of Semmelweis University in Budapest, Hungary, were identified. Semmelweis University is one of the Hungarian COVID-19 centers specializing in the treatment of severe COVID-19 cases in pregnant patients. As a result, the university encountered a higher number of such cases, and nearly all fatal cases involving pregnant COVID-19 patients in Hungary were treated at our institution. The SARS-CoV-2 virus positivity was confirmed using real-time quantitative polymerase chain reaction (RT-q-PCR) technology. Sampling was completed with nasopharyngeal COVID-19 swabs from the upper respiratory tract.

Two authors, DS and BM, independently collected the data from the hospital information system (e-MedSolution; Egészséginformatikai Szolgáltató és Fejlesztési Központ, Budapest, Hungary) of Semmelweis University. A total of 10 pregnant patients with fatal COVID-19 infections were identified and enrolled, and an additional 15 and 20 patients, who survived the disease, were enrolled into two control groups. The control groups were defined as follows: (1) pregnant patients who experienced severe symptoms and required respiratory support (either invasive or non-invasive) but survived COVID-19 disease (*n* = 15); (2) COVID-19 patients who were hospitalized but did not require respiratory support, only closer medical surveillance (*n* = 20). From the patients enrolled in the study, all available NLR values and comorbidities were collected from documentations.

### 2.2. Data Collection

The data were collected during the years 2021 and 2022, which corresponded to the 3rd and 4th waves of the COVID-19 pandemic in Hungary. Initially, all pregnant patients who experienced fatal outcomes at Semmelweis University between 2021 and 2022 were selected for the study. To form the first control group, comprising patients who required respiratory support, individuals were selected between February 2021 and April 2021. For the second control group, 20 pregnant patients who did not require respiratory support due to their SARS-CoV-2 infection and had at least three laboratory results from peripheral blood were randomly chosen from the online medical database. 

In each selected group, over 70% of the patients were from the 3rd wave of the Hungarian COVID-19 pandemic, suggesting likely infection with the beta variant (B.1.351) of the SARS-CoV-2 virus, as the beta variant was the most dominant during the 3rd wave, while the remaining cases were possibly linked to the delta (B.1.617.2) variant.

### 2.3. Statistical Methods

After obtaining the neutrophil and lymphocyte values, their quotient was taken and measured between groups with the help of IBM SPSS Statistics 29. The measured variables were presented in the tables in the format of mean ± standard deviation (SD). The Kolmogorov–Smirnov test was used to determine if the numerical data matched the normality distribution. Variables were compared using Wilcoxon rank-sum test and Fisher’s exact test. Differences were considered statistically significant at *p* < 0.05 both in the Kolmogorov–Smirnov test at the determination of probability and later in other statistical methods. All *p*-values were adjusted using the Holm method [23].

Receiver operating characteristic (ROC) analyses were also carried out to test the sensitivity and specificity of NLR. The ROC analyses were performed in the R for Windows environment (version 4.2.3; R Foundation for Statistical Computing, Vienna, Austria) using the pROC R-library (v1.18.0). The coordinates for the best threshold were identified using Youden’s J statistic. Moreover, the effect of various clinical characteristics on NLR values was investigated. The latter analysis was performed using ANCOVA and mixed-effect (R library nlme, version 3.1-163) models.

## 3. Results

**Figure 1:**
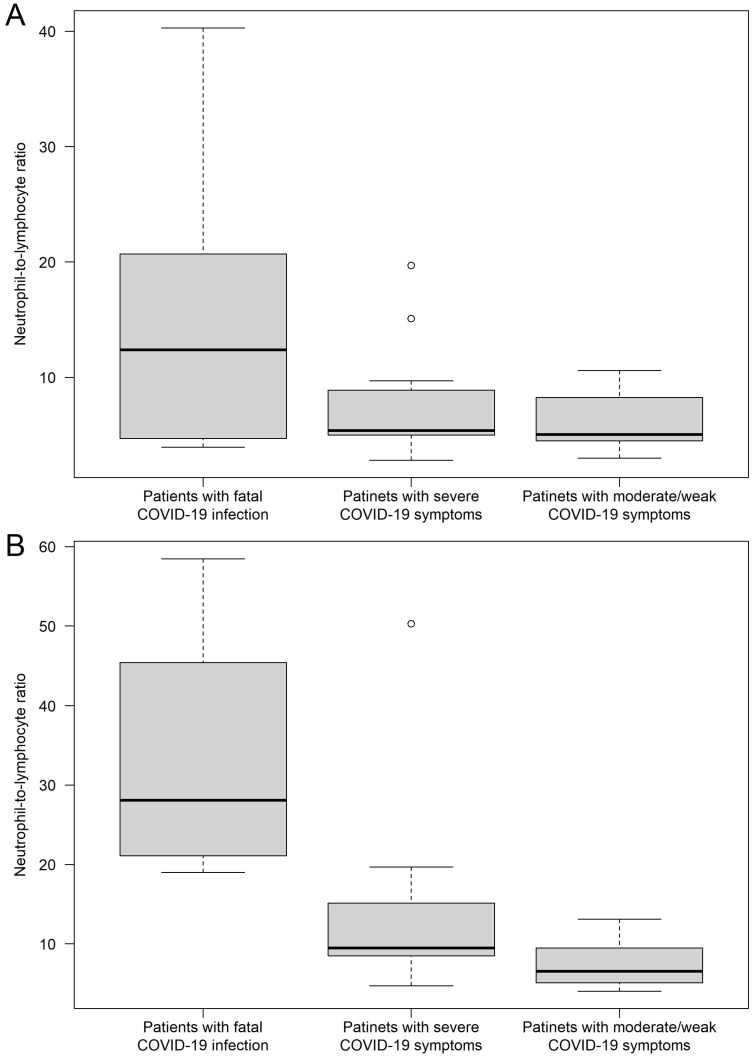
The neutrophil-to-lymphocyte ratios (NLRs) are presented for three groups, including the values on the first day of hospital admission (**A**) and the highest measured value (**B**). Hollow circles represent outliers (greater/lower 1.5 times the interquartile range above/below the upper/lower quartile).

The 45 patients were divided into the three groups, as described above. Age, gestational age when the infection started measured in weeks, first-day NLR values, and peak NLR values of the study participants are presented in Table 1. To better visualize the data, it is also available in the form of a box plot, which presents both the NLR data for day one and the peak NLR (Figure 1).

To compare the groups, we used the Wilcoxon rank-sum test, which initially indicated that there were no significant differences in age and gestational age at the time COVID-19 was contracted. The comparisons of the groups revealed that although the NLR values from the first day of the hospitalization were higher in those patients who later lost their lives (14.56 ± 11.34), no significant difference could be justified neither in the case when comparing to those patients who needed ventilation support and recovered (7.66 ± 4.62; *p* = 0.3926) nor when comparing to moderate COVID-19 cases (5.97 ± 2.23; *p* = 0.1288). In the case of the peak NLR values, the NLR values of the fatal cases were significantly higher, compared to both control groups. Fatal COVID-19 cases had a peak NLR value of 33.77 ± 14.03, while the ventilated but surviving group had an NLR value of 13.35 ± 11.43 (*p* = 0.0006). Furthermore, the same could be observed during the comparisons of the fatal and non-ventilated groups (peak NLR: *p* < 0.0001; Table 2). It must be also highlighted that no difference could be justified between the first-day NLR values of the two control groups, while the peak values were significantly higher in the ventilated group (*p* = 0.0143; Table 1 and Table 2).

To gather more data, we also collected information on the number of comorbidities in the patients, which is presented in Table 3. As observed in the table, 50% of the patients who later deceased due to the disease had no comorbidities. In the ventilated group, this percentage was 53%; whereas, in the group that did not require ventilation support during their hospital admission, this ratio was even higher at 75%. Notably, in the latter group, none of the patients had more than one comorbidity.

**Figure 2:**
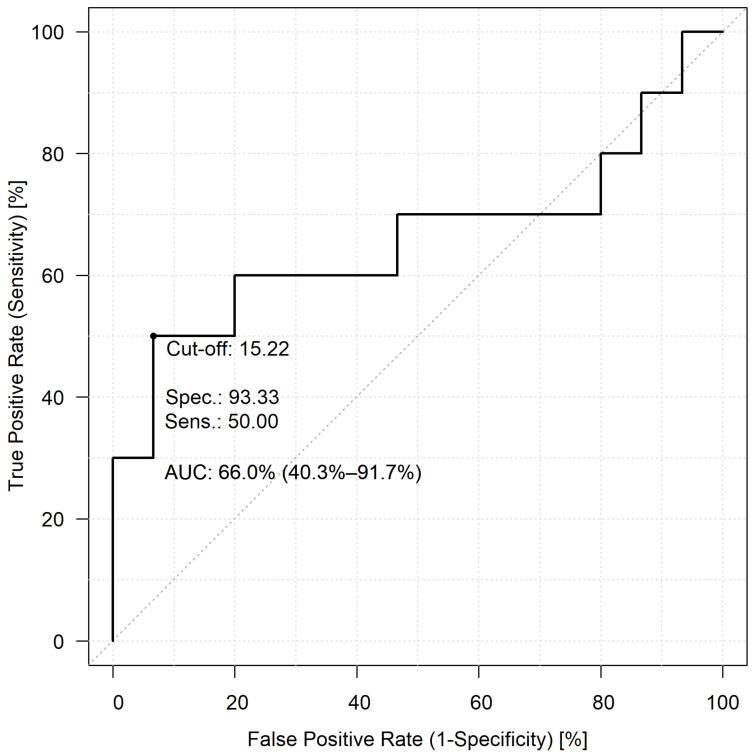
The predictive capability of first-day NLR values in patients who lost their life to COVID-19 infection, compared to those patients who needed mechanical ventilation.

**Figure 3:**
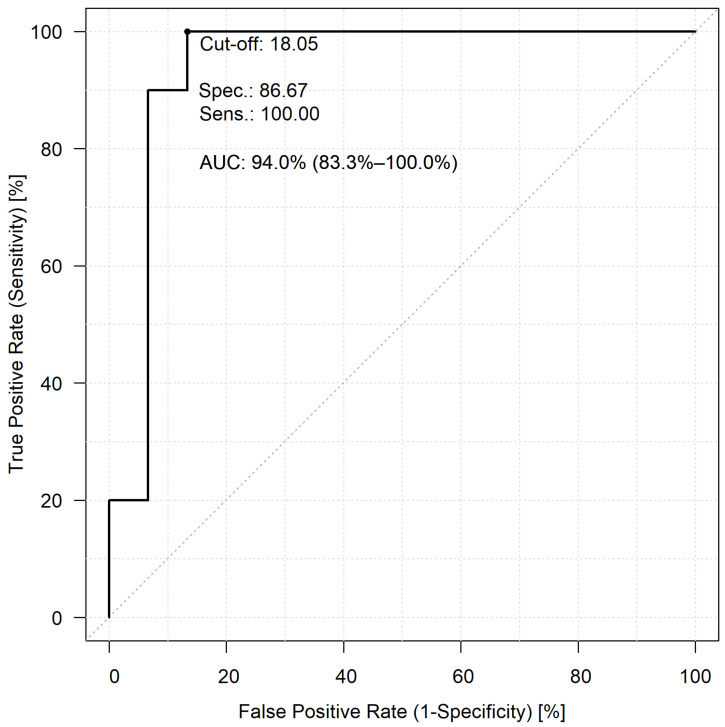
The predictive capability of the highest NLR values in patients who lost their life to COVID-19 infection, compared to those patients who needed mechanical ventilation.

To investigate the predictive potential of the two NLR values and whether they can be used to early identify potential lethal COVID-19 cases, ROC analyses were performed. In this comparison, patients who faced fatal outcomes (Group 1) and those who needed ventilation support therapy (Group 2) were included. For the first-day NLR values, a cut-off value of 15.22, a sensitivity of 50%, a specificity of 93.33%, and an AUC value of 66.0% were found (Figure 2). The comparisons of the peak NLR values revealed a cut-off value of 18.05 with 100% sensitivity and 86.67% specificity, respectively, and the AUC value of the second ROC analysis was 94.0% (Figure 3).

It was investigated whether any of the clinical parameters had an effect on NLR values. Two types of models were constructed for both first-day and peak NLRs. First, an ANCOVA model was used to explore the effects of age, gestational week when COVID-19 occurred, and the number of comorbidities. The analysis revealed that neither age, gestational week, nor the number of comorbidities had a significant impact on NLR values (Table 4). The explanatory power of these models for the NLR was weak, accounting for less than 5% of the variance in both first-day and peak NLR values.

Second, mixed-effect models were constructed to assess whether the number of comorbidities introduced additional variance to the NLR values. The findings indicated that no additional variance was introduced, neither for the first-day NLR nor for the peak NLR values (Table 5).

## 4. Discussion

Neutrophil-to-lymphocyte ratio and its elevation is a widely studied predictive marker in COVID-19: Damar Çakırca et al. collected retrospective data from COVID-19 patients in 2021 and found that NLR is an effective marker for predicting pneumonia in SARS-CoV-2-infected patients [24]. Fois et al. found that NLR values are significantly higher in patients who did not survive COVID-19 compared to those who did. The fact that the measurement of the laboratory values needed for NLR is widespread and relatively inexpensive makes it perfectly suitable to be used for prediction all over the world, even in developing countries [25,26]. 

NLR values are not only elevated in COVID-19 but also in physiological, normal pregnancies [27], and furthermore, in pregnancy-related diseases as well. For instance, NLR values were first evaluated in pre-eclampsia in 2014 by Oylumlu et al., and it was found that NLR values may be useful in the risk stratification of pre-eclampsia [28]. In more recent studies, Thombare et al., in their 2023 case-control study, also found that the NLR was significantly higher in women who developed pre-eclampsia during their pregnancies compared to NLR values measured in healthy control groups [29]. Cui et al. published a retrospective study in 2023 that evaluated the NLR’s predictive value in pre-eclampsia patients. However, in that article, the NLR was used as a predictive marker for liver and coagulation factor dysfunctions. The study involved 320 pregnant patients, and the results indicated that an NLR value greater than 3.7 could potentially be a sign of organ dysfunction in pre-eclamptic patients [30]. In recent years, a large number of further studies have been published on the association between elevated NLR values and pregnancy-related conditions such as gestational diabetes mellitus and pregnancy-induced hypertension [31,32,33]. 

Our research group previously reported the case of a 33-year-old pregnant kidney transplant recipient patient. During her COVID-19 illness, her NLR values were elevated, she required non-invasive respiratory support, and she exhibited pre-eclamptic symptoms [34]. After reporting this interesting case, our focus shifted to researching NLRs in pregnant COVID-19 patients. Our current report reveals that pregnant women who succumbed to COVID-19 had higher NLR values on the first day and significantly higher peak NLR values compared to those who either required mechanical ventilation or did not need ventilation support. It is important to note that while there was a significant difference between the fatal-outcome group and the other two groups, there was no significant difference between the ventilated and non-ventilated groups in terms of first-day NLR values. However, a significant difference did exist regarding the peak NLR values.

Our hypothesis is that monitoring NLR values could help physicians initiate potential treatment methods earlier [35,36,37]. We would like to emphasize the most significant finding of our study: Even though we cannot predict when a patient will reach their peak NLR value, if extremely high NLR values (as determined by our ROC analysis with a threshold of 18.05) are measured in pregnant COVID-19 patients at any point during their hospital admission, it is imperative that the patient be closely monitored. This elevated NLR value could potentially indicate a severe disease outcome. It should also be mentioned that, although the difference was not statistically significant in our analysis, if a pregnant patient’s NLR values are high on the first day of hospital admission, she should be monitored more closely. Physicians should also be well-prepared for the patient’s potential worsening state.

Even though the NLR value is a useful marker for detecting fatal outcomes, differentiating between surviving patients with and without ventilation is not feasible. In the ventilated group, we observed higher values on the first day of hospitalization, and the mean of the maximums was also higher. However, these results are not significant, likely due to the wide range (high variance) of measured NLR values. These data suggest that NLR values are suitable for differentiating critical cases from others, but they are not useful markers for predicting which patients will require ventilation support and which patients will not.

### Strengths and Limitations

We have reported comparisons of NLR data from three groups on two occasions. Despite the low number of cases, this study underscores the potential prognostic significance of NLR value measurements in pregnant COVID-19 patients. Semmelweis University, as a central hospital in Hungary’s COVID-19 healthcare system, allowed us to present NLR values from 10 pregnant mothers who lost their lives due to the infection. While 10 cases may be relatively high compared to other studies, we encourage fellow researchers who have encountered these unfortunate situations to publish NLR values or other potential markers. This collective effort can aid in reducing the number of such fatalities and contribute to the fight against COVID-19 in pregnant patients.

It is important to note that among the patients who died due to COVID-19, there were more comorbidities present, which could have also contributed to the further elevated NLR levels in this group. It is also important to note that, due to the limited number of patients included, ROC statistics may be less reliable in this context.

Since the data presented in this study were collected retrospectively, it was not feasible to perform analyses on specific COVID-19 variants. The typification of COVID-19-infected patients was conducted by the Hungarian Public Health Center. The authors do not have access to these data due to Hungarian law; thus, our conclusions regarding which variant infected each patient were based on national statistics, which identified the dominant variant at the time of infection.

## 5. Conclusions

Our research has revealed that NLR values are valuable for detecting potentially critical and fatal cases of COVID-19, even on the first day of hospital admission. Early identification of elevated NLR values may assist physicians in more closely monitoring these patients and initiating appropriate treatment sooner. It is also essential to emphasize, based on our findings, that NLR values are not effective markers for predicting which patients will require ventilatory support. We encourage other researchers to publish their findings on the same subject, which could potentially lead to the inclusion of a larger number of patients in future studies.

## Figures and Tables

**Table 1:** The patients’ neutrophil-to-lymphocyte ratio (NLR) values and ages. Groups 1, 2, and 3 consist of fatal COVID-19 cases, patients who needed mechanical ventilation (either invasive or non-invasive), and patients who were hospitalized but did not need mechanical ventilation, respectively. The data are presented in the table in the format of mean ± SD; after that, in the brackets, the minimums, maximums, and medians are presented. As the presented data show, the age of the pregnant women in all 3 groups does not differ from each other significantly.

Clinical Characteristic	Group 1 (*n* = 10)	Group 2 (*n* = 15)	Group 3 (*n* = 20)
Age (years)	34.60 ± 5.04	31.87 ± 4.90	33.20 ± 7.05
First-day NLR value	14.56 ± 11.34	7.66 ± 4.62	5.97 ± 2.23
Peak NLR during the infection	33.77 ± 14.03	13.35 ± 11.43	7.54 ± 2.86
Gestational age when the infection started (weeks)	31.30 ± 3.09	30.27 ± 5.46	31.05 ± 4.74

**Table 2:** The comparisons’ adjusted *p*-values of the groups presented in Table 1; *p* < 0.05 was accepted as a significant difference.

Clinical Characteristic	Groups 1 vs. 2	Groups 1 vs. 3	Groups 2 vs. 3
age (years)	*p* = 0.5447	*p* = 1.0000	*p* = 1.0000
first-day NLR value	*p* = 0.3926	*p* = 0.1288	*p* = 0.3926
peak NLR during the infection	*p* = 0.0006	*p* < 0.0001	*p* = 0.0143
gestational age when the infection started (weeks)	*p* = 1.0000	*p* = 1.0000	*p* = 1.0000

**Table 3:** This table provides a breakdown of the number of comorbidities for patient groups, allowing easy comparison and analysis.

Groups	Patients with 0 Comorbidity in Medical History	Patients with 1 Comorbidity in Medical History	Patients with 2 Comorbidities in Medical History
Group 1 (*n* = 10)	5	4	1
Group 2 (*n* = 15)	8	5	2
Group 3 (*n* = 20)	15	5	0

**Table 4:** *p*-values of the ANCOVA models investigating the effect of a few clinical parameters over neutrophil-to-lymphocyte ratio (NLR).

Clinical Characteristic	Model Investigating D1 NLR	Model Investigating Peak NLR
Age (years)	0.5198	0.8520
Gestational week	0.9482	0.4850
Number of comorbidities		
0 vs. 1	0.0776	0.5020
0 vs. 2	0.4077	0.6700
0 vs. 3	0.9138	0.4760

**Table 5:** *p*-values and random effects of the mixed-effect models investigating the effect of a few clinical parameters over neutrophil-to-lymphocyte ratio (NLR).

Clinical Characteristic	Model Investigating D1 NLR	Model Investigating Peak NLR
***p*-Values**		
Age (years)	0.6681	0.3448
Gestational week	0.9742	0.2054
Number of comorbidities		
0 vs. 1	0.1864	0.8583
0 vs. 2	0.6462	0.1610
0 vs. 3	0.7542	0.1649
**Random effect**		
Random effect of NLR values	0.0006	0.0006

## Data Availability

The data supporting the findings of this study can be obtained by contacting the corresponding author upon request.
